# Non Invasive Sensors for Monitoring the Efficiency of AC Electrical Rotating Machines

**DOI:** 10.3390/s100807874

**Published:** 2010-08-23

**Authors:** Farid Zidat, Jean-Philippe Lecointe, Fabrice Morganti, Jean-François Brudny, Thierry Jacq, Frédéric Streiff

**Affiliations:** 1 Univ Lille Nord de France, F-59000 Lille, France; E-Mails: zidat.farid@yahoo.fr (F.Z.); fabrice.morganti@univ-artois.fr (F.M.); jfrancois.brudny@univ-artois.fr (J.-F.B.); 2 UArtois, LSEE, F-62400 Béthune, France; 3 EDF-R&D-Département THEMIS Groupe R25, F-92141 Clamart Cedex, France; E-Mail: thierry.jacq@edf.fr (T.J.); 4 ADEME-SEET Department-20, Av. Du Grésillé BP 90406, F-49004 Angers Cedex 01, France; E-Mail: frederic.streiff@ademe.fr (F.S.)

**Keywords:** magnetic field sensors, efficiency diagnosis, AC electrical motors

## Abstract

This paper presents a non invasive method for estimating the energy efficiency of induction motors used in industrial applications. This method is innovative because it is only based on the measurement of the external field emitted by the motor. The paper describes the sensors used, how they should be placed around the machine in order to decouple the external field components generated by both the air gap flux and the winding end-windings. The study emphasizes the influence of the eddy currents flowing in the yoke frame on the sensor position. A method to estimate the torque from the external field use is proposed. The measurements are transmitted by a wireless module (Zig-Bee) and they are centralized and stored on a PC computer.

## 1. Introduction

The energy consumption monitoring of electric rotating machinery has recently become a major problem. Many companies are undertaking to reduce energy waste, on the one hand to decrease the operating costs and, on the other hand, to contribute to the achievement of environmental commitments. Electric machines are at the heart of the problem since the consumption by electric motors represents over 65% of industry consumption in many European industrialized countries [1]. Applications consuming the major parts of energy are the production of compressed air, pumps and ventilation [2]. It is estimated that significant energy savings can be achieved in these fields with the use of variable speed drives and high performance motors.

The publications on this subject are proliferating with, for instance, the high efficiency induction motors with rotor cage made of copper [3–5]. Other methods uses specific magnetic circuits with non-segmented shifted grain oriented steel laminations, even on machines of small and medium power [6,7]. The growing use of permanent magnet motors [8] also confirms this tendency. The application of standards such as IEC 60034-30Ed1 shows the intention to save electrical energy. However, processes are initially designed for an application defined at a given time. The initial needs may, over the time, change without any modifications of the electrical motors. For example, a motorized pump initially very well chosen might have, a few years later, a low efficiency if its flow is changed. It highlights the importance and necessity of the on-line energy diagnosis of electrical machines and such a diagnosis can concern an entire fleet of electrical machines. Today, some manufacturers offer diagnosis tools directly integrated to their variable speed drives.

However, the machines directly connected to the network constitute the major part of electrical machines and they are not easily diagnosable in an industrial environment [9,10]. The lack of tools dedicated to efficiency diagnosis can be noted. The aim of this paper is to present a non invasive method to diagnose AC motor efficiency. It leads to the conception of a cell which may be installed by non skilled technical staff. Moreover, this method should not, for obvious reasons of organization, security and costs, require opening the terminal box. The cell, a small device placed near the machine, must be able to give accurate information on the consumed energy without measuring the voltage or the speed. From a technical standpoint, the information that the cell has to transmit makes it possible to achieve a complete energy diagnosis: the working time, the temperature, the number of starts, the effective torque, the power and so the speed and, at last, the efficiency. The major impediment consists in determining the torque without any access to the machine voltage and, if possible, without current measurement [11]. The existing methods for determining the torque on-line require knowing the supply voltage or the motor parameters or the rotation speed [12,13]. Finally, the cell must also be capable of operating autonomously for several months.

The presented method is based on the measurement of the external magnetic flux emitted by the motor. Thus, the paper first presents how, from the external magnetic field radiated by the motor, it is possible to separate the effects generated by the straight sections of coils embedded in the slots from the effects generated by the end-windings. Emphasis is placed on the type of sensors used and how they must be placed around the motor. Finite element models make it possible to validate and to extend the results. As the yoke constitutes a shield for the field emission, series of experiments coupled with FE simulations make it possible to evaluate its impact on the radiated field.

Then, the paper describes the information, which can be determined from the external field measurement. A method to estimate the induction motor torque is given. An experimental validation is proposed with an industrial 11kW induction motor machine, highlighting the method accuracy.

Finally, the paper shows how the information collected can be transmitted with a wireless system, working autonomously for several months. The simplicity of the magnetic flux sensors promotes their own energy consumption and provides to the application a real interest.

## 2. Decoupling of the External Magnetic Field Components

### 2.1. External magnetic field around an AC electrical motor

**Figure 1.**
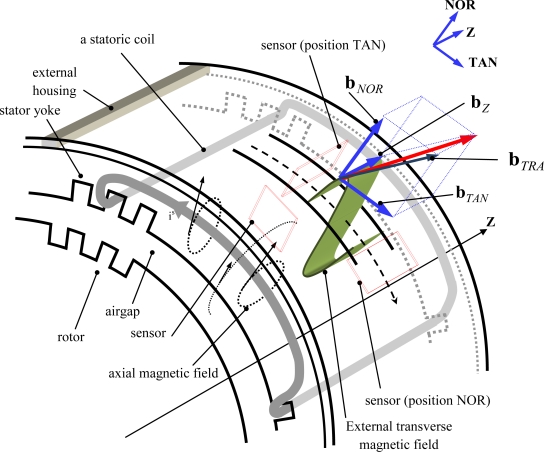
External magnetic field emitted by an AC machine.

The external magnetic field radiated by a 3-phase *p* pole pair machine can be characterized by two components: a **b***_z_* axial component oriented along the motor axis **Z** and a **b***_TRA_* transverse component as shown in Figure 1. The transverse component has, if a cylindrical frame is considered, 2 projections: a **b***_NOR_* normal component and a **b***_TAN_* tangential component respectively oriented with the axes **NOR** and **TAN** [14].

**Figure 2.**
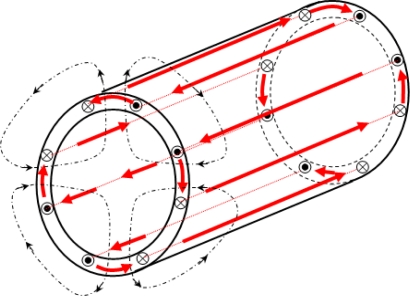
Eddy currents in the stator yoke.

The major difficulty when external flux is measured is to separate the influence of the currents flowing in the end-winding connections from the effect of the air gap field. Simplified approaches assimilate the end windings field to the current flowing in the coils and the transverse field to the air gap field. Nevertheless, the phenomena are more complex. Indeed, the transmission of the field from the air gap to the outside of the motor requires the crossing of the sheet stack and then the yoke. Previous studies [15–17] show that the yoke is responsible for a major mitigation. As it is made of one piece, contrary to the laminated stator magnetic circuit, eddy currents flow in the yoke, creating a magnetic field, which the effects are opposed to the main field (Figure 2). As a consequence, the external field is attenuated and phase-shifted. It will be shown that those effects are dependent on both the machine load state and the magnetic circuit saturation, making the study a little more delicate.

In the paper, right superscripts “*a*” or “*tr*” indicate if the considered variable result from the end-winding field (as “axial field”) or from the air gap field (as “transverse field”). A subscript is added in brackets to specify the rank of the harmonic considered and “*g*” is added if the variable concern the air gap.

Thus, for example, $b_{\mathrm{TAN},(1)}^{a}$ corresponds to the fundamental external normal component generated by the end-windings. Sinusoidal time variables are denoted *x*(*t*) = *x̂* cos(ω*t* + φ) where $\hat{x}=X\sqrt{2}$. Capitals designate complex quantities. *ω* is the angular frequency of the fundamental variables.

### 2.2. Sensors used for magnetic field measurement

Different types of sensors are available to measure magnetic fields of low magnitude [18]. Hall effect sensors or magneto-resistive probes are quite common. The sensors used in this paper are made of a coil with several turns. They provide three main advantages: first, exploiting the information by measuring the coil voltage is relatively simple. No specific associated electronic device is required; just an amplification of the signal is done. Then, the sensor consumption is limited to that of the amplifier. It has a significant importance in case of embedded and autonomous sensors. Finally, such a probe is easy to manufacture with different forms [19].

**Figure 3.**
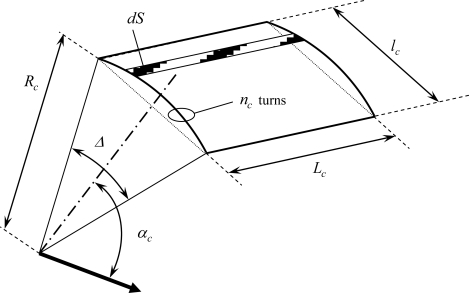
Sensor model.

Let us consider a sensor made of a rectangular coil of *L_c_* length and *l_c_* width, placed against the motor yoke (position NOR) at a *R_c_* distance from the motor axis, as shown in Figure 3. *L_c_* is small relatively to the machine effective length so that the field in Z direction can be considered as uniform. *Δ* and *α_c_* are respectively the angular sensor opening and the position of the sensor axis compared with the phase 1 axis. (we neglect for this demonstration phase shifts introduced by the magnetic circuit and the yoke). Because of the sensor dimensions, *l_c_* ≈ *R_c_Δ* even if the sensor is flat.

An idealized model is assumed: the transverse flux density $b_{\mathrm{NOR},(1)}^{\mathrm{tr}}$ is supposed to be sinusoidal outside the machine and reduced of a *K* factor:

(1) $$b_{\mathrm{NOR},(1)}^{\mathrm{tr}}(t)=K{\hat{b}}_{\mathrm{NOR},(1)}^{\mathrm{tr}}\;\text{cos}(\omega t-{p\alpha }^{s})$$

The elementary flux density $\psi_{\mathrm{NOR},(1)}^{\mathrm{tr}}$ through *dS* = *d*(*L_c_l_c_*) = *L_c_d*(*l_c_*) ≈ *L_c_R_c_dα^s^* is given by:

(2) $$d\psi_{\mathrm{NOR},(1)}^{\mathrm{tr}}={\mathrm{KbL}}_{c}R_{c}{d\alpha }^{s}$$

Integration of (2) leads to Expression (3):

(3) $$\psi_{\mathrm{NOR},(1)}^{\mathrm{tr}}=\int_{\alpha^{s}-\frac{\Delta }{2}}^{\alpha^{s}+\frac{\Delta }{2}}{K\hat{b}L}_{c}R_{c}\;\text{cos}\left(\omega t-{p\alpha }^{s}\right){d\alpha }^{s}=\frac{{K\hat{b}L}_{c}R_{c}}{p}\text{sin}\left(p\frac{\Delta }{2}\right)\text{cos}\left(\omega t-{p\alpha }^{s}\right)=\hat{\psi }\;\text{cos}\left(\omega t-{p\alpha }^{s}\right)$$

As a consequence, $\psi_{\mathrm{NOR},(1)}^{\mathrm{tr}}$ depends on the distance *R_c_* and the dimensions of the sensor. Large *L_c_* increases the flux seen by the coil and *Δ* has to be well chosen because sin(pΔ/2) can be null. The sensor dimensions have to be adjusted to the pole pair number. It is especially the case for low speed motors for which *p* is high. These wounded sensors are particularly interesting because they have the advantage of deriving the external flux. The low amplitude of *k* rank flux density harmonics is multiplied by*kω*.

Practically, for experiments presented in this paper, the coil voltage is directly analyzed with a Brüel & Kjaer spectrum analyzer. This type of analyzer, rather used for noise and vibration studies, has the interest to treat low amplitude signals.

**Figure 4.**
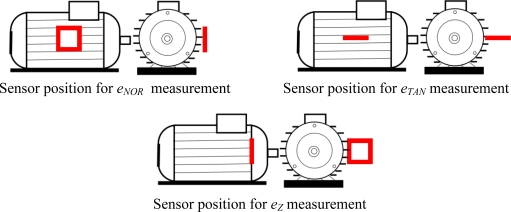
Sensor positions.

Figure 4 shows more clearly the three positions of the sensor to measure the components **b**_*Z*,(*k*)_, **b**_*NOR*,(*k*)_ and **b**_*TAN*,(*k*)_. It is essentially the fundamental component (50 Hz, k = 1) which is measured. The coil voltage is denoted *e* with superscript and subscripts previously defined.

### 2.3. Distinction external magnetic field components: influence of the end-windings

**Figure 5.**
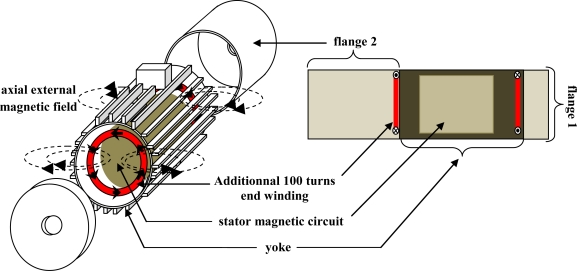
Artificial end-windings.

**Figure 6.**
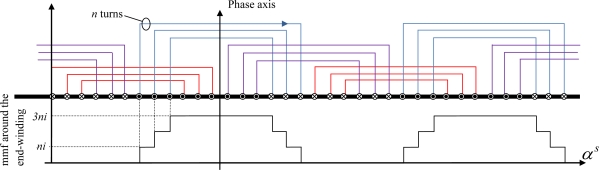
mmf generated by one supplied phase.

In normal operation, it is difficult to separate the effects of end-winding heads from those of wires placed in the slots. A solution is to add artificial end-windings over the real ones (Figure 5). They are made of two additional coils, supplied by a sinusoidal current, to generate a pulsing magnetic field. They can not obviously create a rotating field otherwise the comparison with the 3 phases supply would be flawed. It is possible to partially circumvent this problem by supplying a single phase machine: the mmf, and therefore the flux density, are pulsating with a constant magnitude when all the phase coils are considered (Figure 6). Equality of ampere-turns makes it possible to define the current injected in the artificial coils. The measurement was performed on a 11 kW 2 pole pair induction machine with a wounded rotor.

**Figure 7.**
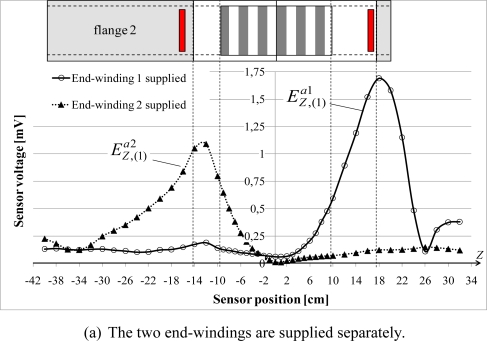

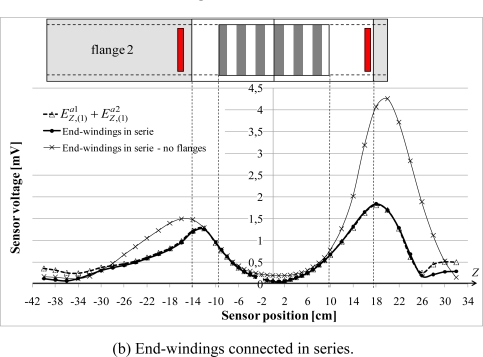
Measurement of the axial field component along the *Z* axis: variations of the $E_{Z,(1)}^{a1}$ and $E_{Z,(1)}^{a2}$ sensor fundamental voltages obtained with the artificial end-windings.

The axial field was observed experimentally when the additional coils are fed separately and then when they are connected in series. Figures 7a and 7b show the $E_{Z,(1)}^{a}$ variation along the *Z* axis (the abscissa 0 is the middle of the magnetic circuit) in two configurations:

- When the coils are fed separately (Figure 7.a), $E_{Z,(1)}^{a1}$ and $E_{Z,(1)}^{a2}$ are measured
- When connected in series, the coils generate a field so that $E_{Z,(1)}^{a}$ is measured. The Figure 7b also shows the sum of $E_{Z,(1)}^{a1}$ and $E_{Z,(1)}^{a2}$ obtained with and without the flanges.

Figure 7a shows that each of the two end-windings generates an axial field concentrated at the edges of the stator yoke. $E_{Z,(1)}^{a2}$ is 30% lower than $E_{Z,(1)}^{a1}$, that is to say along the longer flange (The size of the two flanges is different because of the ring-brush device). Figure 7b shows that the $E_{Z,(1)}^{a1}+E_{Z,(1)}^{a2}$ sum and $E_{Z,(1)}^{a}$ are overlapped. It means that the phenomena generated by each end-winding can decoupled. The minimum value of $E_{Z,(1)}^{a}$, called “neutral magnetic point”, corresponds almost to the magnetic stator circuit middle.

When the flanges are removed, the variation is similar. Nevertheless, the flanges attenuate the radiated field, whether in front of the magnetic circuit (that is clearly visible on the magnetic neutral point) or around the end-windings.

**Figure 8.**
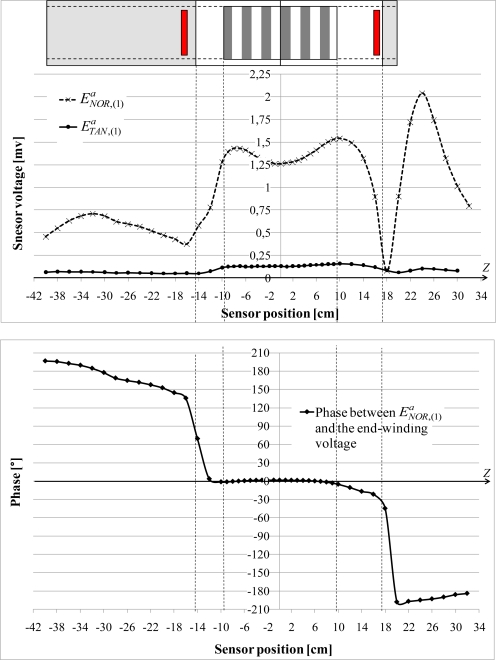
Variations of the normal $E_{\mathrm{NOR},(1)}^{a}$ and tangential $E_{\mathrm{TAN},(1)}^{a}$ fundamental components due to the end-windings with *Z*.

Figure 8 shows the variations, always along the *Z* axis, of the normal $E_{\mathrm{NOR},(1)}^{a}$ and tangential $E_{\mathrm{TAN},(1)}^{a}$ fundamental components measured by the sensor when the artificial end-windings are series connected. For the two measurements, the sensor is placed against the yoke. The $E_{\mathrm{TAN},(1)}^{a}$ component is about 10 times lower than $E_{\mathrm{NOR},(1)}^{a}$. This result is logical because of the coil orientation. Furthermore, the $E_{\mathrm{NOR},(1)}^{a}$ component has three characteristic zones:

In front of the magnetic circuit, $E_{\mathrm{NOR},(1)}^{a}$ varies of 25% between the edges and the middle of the sheet pack.

The second part concerns yoke surfaces which are not in contact with the magnetic circuit: the external decreases sharply, reaching a null value. The sensor voltage phase compared to the coil voltage changes of sign.

Finally, the external field presents a peak in front of the motor flanges, more or less high depending on the flange length.

**Figure 9.**
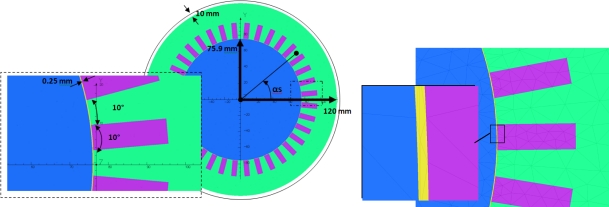
Motor EF 3D geometry and mesh.

**Figure 10.**
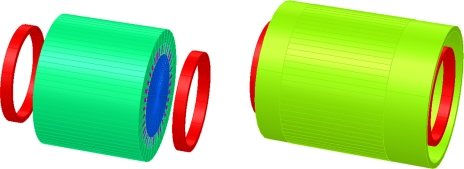
Induction motor with additional end-windings.

The motor used for the tests was simulated with the finite element software Opera 3D. Figure 9 shows both the model and meshing. The simulation can take into account the permeability of the iron and the yoke. The simulation conditions are the same as for the previous experiment: the rotor is static and its winding is not short-circuited,additional end-windings are added (Figure 10).

**Figure 11.**
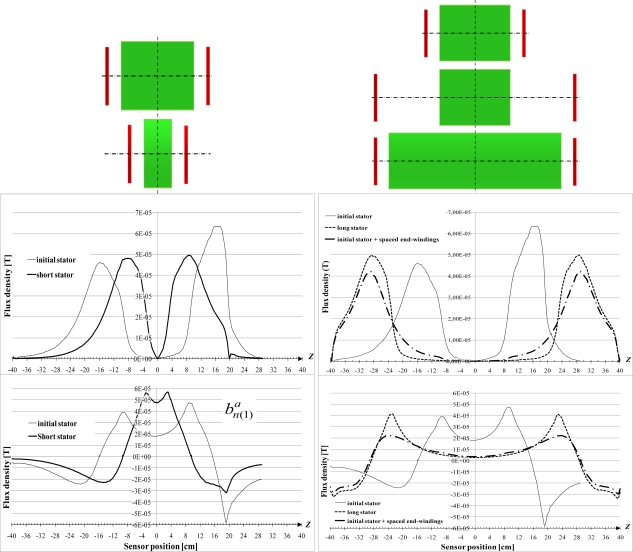
Variations of the normal $B_{\mathrm{NOR},(1)}^{a}$ and tangential $B_{Z,(1)}^{a}$ fundamental components with *Z* - Influence of the stator length and of the end-winding spacing.

The eddy currents in the yoke and the saturation are first neglected. Figure 11 shows that the field calculated outside the motor has the same variations as found experimentally. Then, cases of long and short stators are also highlighted as well as the case of end-windings far from the magnetic circuit. For those simulations, both the stator and rotor magnetic circuits are changed and the yoke and flanges are modified to be still well adapted.

In the three cases, the $B_{Z,(1)}^{a}$ axial fundamental component due to the artificial end-windings has peaks in front of the end-windings and, whatever the configuration, $B_{Z,(1)}^{a}$ is null at the neutral magnetic point. Meanwhile, at this neutral magnetic point, the $B_{\mathrm{NOR},(1)}^{a}$ normal fundamental component due to the artificial end-windings presents a variation which tends to be constant. It is particularly true when the end-windings are much spaced. The magnetic circuit length has an effect on the $B_{\mathrm{NOR},(1)}^{a}$ magnitude in the middle of the sheet pack and it changes the length where $B_{Z,(1)}^{a}=0$.

### 2.4. External field in normal operation at no load

**Figure 12.**
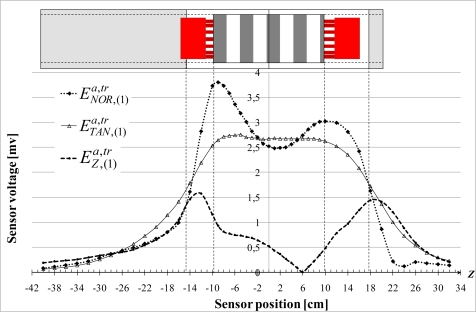
Variations of the normal, tangential and axial fundamental components $E_{\mathrm{NOR},(1)}^{a,\mathrm{tr}}$, $E_{\mathrm{TAN},(1)}^{a,\mathrm{tr}}$ and $E_{Z,(1)}^{a,\mathrm{tr}}$ due to the end-winding field and the air gap field.

Next step of the analysis consists in measuring the external field to quantify the effects of the end-windings and the air-gap flux density transmission. Figure 12 shows the variations of the normal, tangential and axial fundamental components $E_{\mathrm{NOR},(1)}^{a,\mathrm{tr}}$, $E_{\mathrm{TAN},(1)}^{a,\mathrm{tr}}$ and $E_{Z,(1)}^{a,\mathrm{tr}}$ along *Z* when the machine is supplied by the grid. It works at no load with the rotor short-circuited. The $E_{\mathrm{NOR},(1)}^{a,\mathrm{tr}}$ component is distinctly affected by the field of end-windings, especially just next to the borders of the stator. Contrariwise, the tangential component is almost constant in front of the stator magnetic circuit. This is an important result because it means that $B_{\mathrm{TAN},(1)}^{\mathrm{tr}}$ is not disturbed by the axial field ( $E_{\mathrm{TAN},(1)}^{a,\mathrm{tr}}\approx E_{\mathrm{TAN},(1)}^{\mathrm{tr}}$) and an image of the air gap flux density can be obtained. The variation could have been different because the end-windings are curved. Note that normal and tangential components have similar magnitudes: $E_{\mathrm{TAN},(1)}^{a,\mathrm{tr}}\approx E_{\mathrm{NOR},(1)}^{a,\mathrm{tr}}$.

The $E_{Z(1)}^{a,\mathrm{tr}}$ component variation is quite similar to the variations obtained with the artificial end-winding. Peaks are observed near the end-windings but the difference between the two sides is not discernible. It can also be observed that the magnetic neutral point $E_{Z,(1)}^{a,\mathrm{tr}}=0$ is displaced by 6 cm.

**Figure 13.**
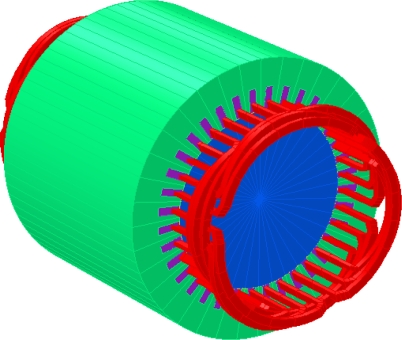
Model of the induction machine magnetic circuits and end-windings.

**Figure 14.**
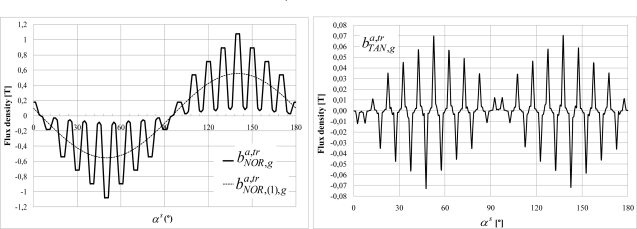
Variations along two poles of the normal and tangential air gap flux density components $b_{\mathrm{NOR},g}^{a,\mathrm{tr}}$, $b_{\mathrm{NOR},(1),g}^{a,\mathrm{tr}}$ and $b_{\mathrm{TAN},g}^{a,\mathrm{tr}}$.

As for the study of the end-winding influence, the machine has been simulated with Opera 3D. The rotor is static and its winding is not short-circuited. Figure 13 shows the model of the magnetic circuits and the end-windings. Figure 14 shows the variations of the normal air gap flux density ( $b_{\mathrm{NOR},(1),g}^{a,\mathrm{tr}}$ and $b_{\mathrm{NOR},g}^{a,\mathrm{tr}}$) and the variations of the tangential air gap flux density $b_{\mathrm{TAN},(1),g}^{a,\mathrm{tr}}$ along two poles (*α^s^* between 0 and 180°). $b_{\mathrm{TAN},(1),g}^{a,\mathrm{tr}}$ is low with peaks in front of the tooth borders. The $b_{\mathrm{NOR},(1),g}^{a,\mathrm{tr}}$ variation between teeth and slots is well marked.

**Figure 16.**
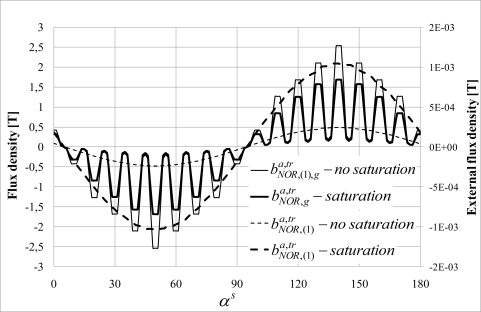
Variations of $b_{\mathrm{NOR},g}^{a,\mathrm{tr}}(\alpha^{s})$ and $b_{\mathrm{NOR},(1),g}^{a,\mathrm{tr}}(\alpha^{s})$ at the neutral magnetic point with and without saturation.

The variations, with *Z*, of the normal, tangential and axial fundamental components $B_{\mathrm{NOR},(1)}^{a,\mathrm{tr}}$, $B_{\mathrm{TAN},(1)}^{a,\mathrm{tr}}$ and $B_{Z,(1)}^{a,\mathrm{tr}}$ are presented at Fig 16. Cases of short and long magnetic circuits are shown, as well as the influence of the end-winding spacing. It confirms that, when the stator becomes longer:

$B_{\mathrm{TAN},(1)}^{a,\mathrm{tr}}$ tends to be constant in front of the magnetic circuits and it is not influenced by the end-windings. Whatever the magnetic circuit length and the end-winding spacing, $B_{\mathrm{TAN},(1)}^{\mathrm{tr}}$ at the neutral magnetic point keeps the same value.

$B_{\mathrm{NOR},(1)}^{a,\mathrm{tr}}$ is, as for the simulation done with artificial end-windings, influenced by the end-windings. They generate a $B_{\mathrm{NOR},(1)}^{a}$ component which is canalized by the iron and combined with the $B_{\mathrm{NOR},(1)}^{\mathrm{tr}}$ component. As a consequence, the value at the neutral magnetic point depends on the distance between the end-windings. For instance, $B_{\mathrm{NOR},(1)}^{\mathrm{tr}}$ at the neutral point is different for both the short and initial stator when compared to the value obtained with spaced end-windings.

$B_{Z,(1)}^{a,\mathrm{tr}}$ is always null at the middle of the sheet stack and the border effects decrease with the stator length.

The variations are quite similar to those obtained with the pulsating field.

Saturation of the magnetic circuits may have an influence on the external field. 3D EF simulations allow taking into account the saturation of the magnetic circuits. Two simulations have been performed:

- The first neglects the saturation: $B_{\mathrm{NOR},(1),g}^{a,\mathrm{tr}}=1.3\;T$ and the *B*(*H*) magnetic characteristic is linear (μ_r_ = 5,000).
- The second uses a *B*(*H*) curve with μ_r_ = 5,000 for H < 600 Am, and B = 1,7 T if H < 600 Am. Flux density in the air gap is: $B_{\mathrm{NOR},(1),g}^{a,\mathrm{tr}}=0,94\;T$.

Figure 16 shows the $B_{\mathrm{NOR},g}^{a,\mathrm{tr}}$ normal air gap flux density component variations along the airgap. Saturation implies a decrease of the peak value of $B_{\mathrm{NOR},g}^{a,\mathrm{tr}}\;(\alpha^{s})$. However, the $B_{\mathrm{NOR},(1)}^{a,\mathrm{tr}}(\alpha^{s})$ fundamental normal flux density component outside the machine is 4.47 times higher when saturation is taken into account. Variations of the tangential and normal components have the same waveforms when the saturation is taken into account; only their amplitudes are modified.

**Figure 17.**
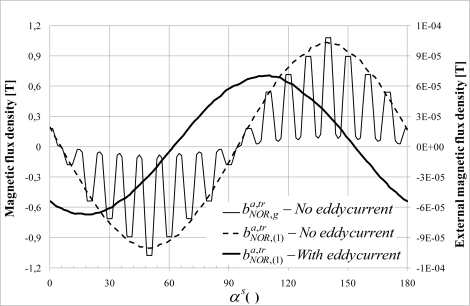
Influence of the eddy currents in the yoke on the normal external field.

The eddy current in the yoke are responsible from a decrease and a phase shift. The Figure 17 shows the variation around the stator of the $b_{\mathrm{NOR},(1)}^{a,\mathrm{tr}}$ normal external flux density component obtained numerically with Opera 3D. In the simulation, the yoke conductivity is 10^6^ S/m. It shows that the eddy currents are responsible on a decrease of 33% and a phase difference of 30°. The same phenomenon (mitigation and shifting) has been observed for the tangential component.

### 2.5. External field in normal operation in load

A 11 kW 2-pole pair induction machine with squirrel cage rotor has been tested in load with a DC machine. It is equipped of a coil in the air gap so that the external magnetic field component can be compared to the air gap field.

**Figure 19.**
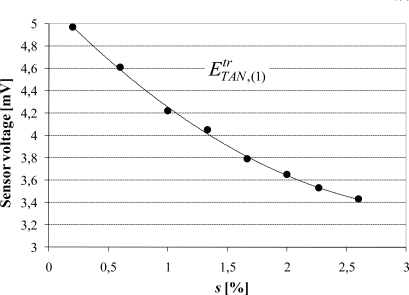
Variations of the fundamental tangential component $E_{\mathrm{TAN},(1)}^{\mathrm{tr}}$ with *s*.

The Figure 19 shows the variations of $B_{\mathrm{NOR},(1)}^{a,\mathrm{tr}}$ and $B_{\mathrm{TAN},(1)}^{a,\mathrm{tr}}$ (measurement points and interpolation) with the $I_{\left(1\right)}^{s}$ fundamental phase current and when the sensor is placed at the neutral magnetic point. Each component is presented for two simple phase voltages: *V^s^* = 220V and *V^s^* = 186V. It allows measuring the saturation influence. As expected, the variation of the normal component depends on the current flowing in the end-windings, which generate the $b_{\mathrm{NOR},(1)}^{a}$. The $B_{\mathrm{TAN},(1)}^{a,\mathrm{tr}}$ variations (compared to the measure at no load) are close from the air-gap flux variations. With the previous measurements, this difference can be explained by both the magnetic circuit saturation and the eddy currents in the yoke.

## 3. Application to a Cell for the Efficiency Diagnosis of Induction Machines

### 3.1. Efficiency diagnosis of AC electrical machines in industrial environments

The information to make up an energy diagnosis is: the working time, the temperature, the number of starts, the effective torque, the power and so the speed and, at last, the efficiency. The major problem consists in determining the torque without any access to the voltage of the machine. The existing methods for determining the torque on-line require to know the supply voltage, the motor parameters or the rotation speed [12]. At last, the cell must also be autonomous for several months.

The external magnetic field measurement with wounded sensors makes possible to determine easily the number of starts and the working time. For the torque estimation, the idea consists in using the external flux variation. A previous study [20] shows that an empirical determination is possible by measuring the stator current variations. However, this method implies to use a current sensor.

As a consequence, in order to simplify the cell and to minimize cost, only the flux variation measurement is exploited with the rated values. As for many simplified models, the electromagnetic torque is supposed to be linear with the slip *s*:

(4) $$\Gamma_{e}=\mathrm{A.s}$$

Then, the sensor is supposed to be placed in position TAN, at the middle of the stator magnetic circuit, in order to measure the fundamental tangential component $E_{\mathrm{TAN},(1)}^{\mathrm{tr}}$. Figure 19 shows the variation of $E_{\mathrm{TAN},(1)}^{\mathrm{tr}}$ with *s*. The variation is almost linear when *s* > 1%, that is to say when the stator current is higher than $I_{\left(1\right)}^{s}>I_{(1),n}^{s}/3$ (The subscripts “0” and “n” denote respectively the working point at no load and the nominal values). So, in this range, it can be written:

(5) $$E_{\mathrm{TAN},(1)}^{\mathrm{tr}}=\alpha \;s+\beta$$

As, for $s\approx 0\;:\;E_{\mathrm{TAN},(1)}^{\mathrm{tr}}=E_{\mathrm{TAN},(1),0}^{\mathrm{tr}}$ and for $s\approx s_{n}\;:\;E_{\mathrm{TAN},(1)}^{\mathrm{tr}}=E_{\mathrm{TAN},(1),n}^{\mathrm{tr}}$, the Equation (5) gives :

(6) $$E_{\mathrm{TAN},(1)}^{\mathrm{tr}}=\frac{E_{\mathrm{TAN},(1),n}^{\mathrm{tr}}-E_{\mathrm{TAN},(1),0}^{\mathrm{tr}}}{S_{n}}S+E_{\mathrm{TAN},(1),0}^{\mathrm{tr}}$$

Thus, *s* is given by:

(7) $$S=S_{n}\frac{E_{\mathrm{TAN},(1),0}^{\mathrm{tr}}-E_{\mathrm{TAN},(1)}^{\mathrm{tr}}}{E_{\mathrm{TAN},(1),0}^{\mathrm{tr}}-E_{\mathrm{TAN},(1),n}^{\mathrm{tr}}}$$

It leads to :

(8) $$\Gamma_{e}={\mathrm{As}}_{n}\frac{E_{\mathrm{TAN},(1),0}^{\mathrm{tr}}-E_{\mathrm{TAN},(1)}^{\mathrm{tr}}}{E_{\mathrm{TAN},(1),0}^{\mathrm{tr}}-E_{\mathrm{TAN},(1),n}^{\mathrm{tr}}}=\Gamma_{e,n}\frac{E_{\mathrm{TAN},(1),0}^{\mathrm{tr}}-E_{\mathrm{TAN},(1)}^{\mathrm{tr}}}{E_{\mathrm{TAN},(1),0}^{\mathrm{tr}}-E_{\mathrm{TAN},(1),n}^{\mathrm{tr}}}$$

The torque determination requires knowing *Γ_e,n_*; its value given on the rating plate can be used. The process is also based on the value of the sensor voltage at no load ( $E_{\mathrm{TAN},(1),0}^{\mathrm{tr}}$) and at the nominal load ( $E_{\mathrm{TAN},(1),n}^{\mathrm{tr}}$).

The $E_{\mathrm{TAN},(1)}^{\mathrm{tr}}$ variation (from no-load to the nominal load) for the 11 kW cage induction machine makes possible to calculate the torque with the described method. Results are compared with the measured torque and the theoretical electromagnetic torque determined with the machine single phase equivalent circuit. Theoretical values are close from the value based on the external flux measurements. The difference does not exceed 15% when $I_{\left(1\right)}^{s}>I_{(1),n}^{s}/3$. It constitutes a crucial point for the torque diagnosis: the torque variations can be observed from a non-invasive measurement. At last, it can be noted that the speed can be determined with Equation (7).

### 3.2. Transmission and processing of the data

The information determined with the previous method makes it possible to achieve an energy diagnosis. The next step is the analysis and the transmission of the data. Ideally, the cell is wireless and must also be able to operate autonomously for at least several months.

The whole system is composed of three parts. First, the cell itself is fixed on the side of the machine, the nearest of the external frame for obvious reasons of flux uptake. Each cell is equipped with a module ZigBit (Meshnetics-Atmel). Its function is to concentrate the collected data and then to transmit them. Then, an interface card receives the data analyzed by a computer. The actual devices allow transmitting the data over several hundred meters. The reception card serves as a hub which processes the data of the AC motors equipped of cells. Making data transmission wireless is energy consuming as well as the frequency of emissions. Advantage of the cells is that they can become a transmitter of data coming from neighboring cells. It is also possible to reduce the distance between the cells and the hub.

Advantages of a wireless cell are numerous. It is less expensive, more flexible and it constitutes a highly reliable alternative for existing wired monitoring and for controlling parameters of electric motors. According to this concept, the wireless sensor network technology should integrate the following requirements: a low power consumption, an unlicensed radio band, a good scalability in order to support large number of nodes, a high flexibility to be simply deployed and to extend the network. Note that a low data rate is sufficient.

The most popular standards for wireless sensor networks are the IEEE 802.15.4 specified by Institute of Electrical and Electronics Engineers (IEEE) and the ZigBee protocol developed by ZigBee Alliance. ZigBee low stack layers (PHY and MAC) are equal to those in IEEE 802.15.4 while high stack layers are designed to support extended networking functionality and to provide simple interface between network and end user applications.

ZigBee standard specifies three different types of nodes that might be present in a ZigBee network: a coordinator, a router and an end device. The device is composed of:

a coordinator, which acts as the “root” node. We can configure the key networking parameters, the network start and the network address assignment;
a end device, which can directly communicate with a coordinator to transfer data from the sensor.

It is the simplest topology possible in a ZigBee network. It corresponds to IEEE 802.15.4 star topology. The end-device structure is made with:

the magnetic sensor;
the temperature sensor integrated on the Zigbit board;
an amplifier (A) made with an integrated amplifier (low consumption);
the ZigBit board, which integrates all the components needed to convert and to send data from sensors;
the power supply equal to 7.2 V.

The coordinator is associated with a simple computer. The ZigBit board is supplied by the USB connection. Atmel’s AVR studio was used to develop the application for the end device and the coordinator based on ZigBeeNet API. The magnetic sensor has analog output; so an ADC converter is necessary. Hardware interface covers physical interface between the sensor and the ZigBit board. By default, the board has three ADC channels where the sensor can be connected. Thus, up to two independent analog sensors may be connected without any hardware modification. The temperature sensor does not need an ADC interface, the I2C bus is used to transfer the data to the Zigbit board. The board allows measuring power consumption of the end device. A simple program is developed for reading data from the magnetic and temperature sensors, converting it to a meaningful value representing the current and transferring said values to the higher layer of the ZigBee stack. This layer forwards its data wirelessly to the coordinator device. The coordinator is simply connected to a PC, which outputs data transferred from the end device. Currently, a simple frontend is used to visualize the data from the end-device to the LCD screen.

## 4. Conclusions

This paper develops a non invasive method for a complete diagnosis of AC electrical machines. Its principle is based on the use of the external magnetic field radiated by the motor. The main difficulty was to understand how the flux is radiated outside the motor. Several experiments and EF simulations make it possible to conclude on the origin of the external field components. The tangential component of the external transverse filed is the most usable because it gives an image of the flux density in the air gap. We have also highlighted the influence of the magnetic saturation of the stator and rotor magnetic circuits. The yoke has been identified has a shield which acts on the phase and the magnitude of the external field. At last, a method to perform an energy diagnosis has been proposed. With the external flux measurement, information as the working time or the number of starts can be easily detected. The real challenge was to propose a method to determine the torque only with the external field measurement. An experimental validation on an industrial 11 kW induction machine highlights the possibility to estimate, with an acceptable accuracy, the torque. Finally, the paper shows how the information collected can be transmitted with a wireless system.

Further work will consist in determining more accurately the torque. Moreover, the complete device could also be used to diagnose the AC motor faults as broken bars for induction machines [21,22]. The wounded sensors are of interest because they make it possible to exploit the external magnetic field harmonics.

## Figures and Tables

**Figure 15.**
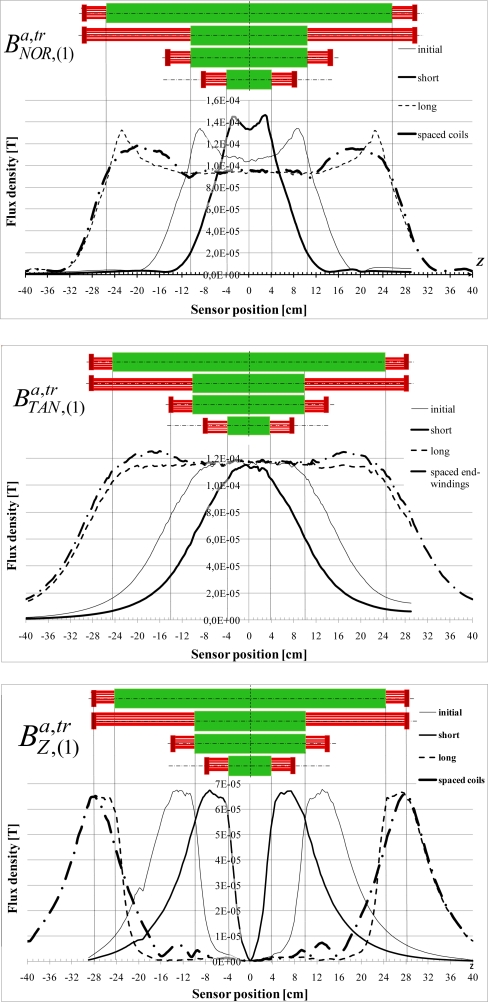
$B_{\mathrm{NOR},(1)}^{a,\mathrm{tr}}$, $B_{\mathrm{TAN},(1)}^{a,\mathrm{tr}}$ and $B_{Z,(1)}^{a,\mathrm{tr}}$ variations with *Z*.

**Figure 18.**
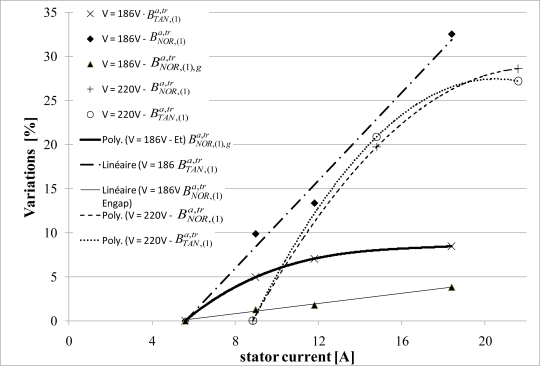
Variations of $B_{\mathrm{NOR},(1)}^{a}$, $B_{\mathrm{TAN},(1)}^{a,\mathrm{tr}}$ and $B_{\mathrm{NOR},(1),g}^{a,\mathrm{tr}}$ with the load.

**Figure 20.**
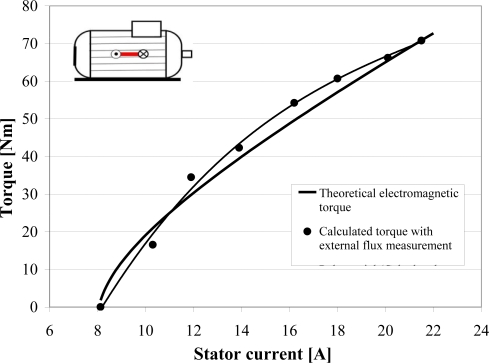
Torque estimation with $E_{\mathrm{TAN},(1)}^{\mathrm{tr}}$ measurement.

**Figure 21.**
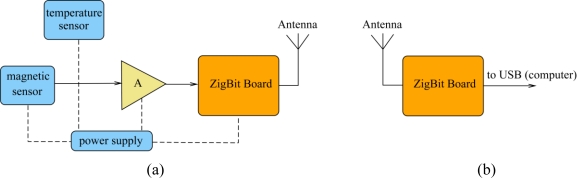
(a) End device. (b) Coordinator with computer.
